# 10 tips on how to take a proper family history in CKD patient care

**DOI:** 10.1093/ckj/sfaf253

**Published:** 2025-08-07

**Authors:** Emilie Cornec-Le Gall, Albertien M van Eerde, Lucile Figueres, Matias Simons, Giovambattista Capasso, Maria Vanessa Perez Gomez, Tom Nijenhuis, John A Sayer, Nikola Zagorec, Roman-Ulrich Müller, Jan Halbritter

**Affiliations:** Department de Néphrologie, Hémodialyse et Transplantation Rénale, Centre de référence MARHEA, Filière ORKID, CHRU Brest, Brest, France; University Brest, Inserm, UMR 1078, GGB, Brest, France; Department of Genetics, University Medical Centre Utrecht, Utrecht, the Netherlands; Nantes Université, CHU Nantes, INSERM, Center for Research in Transplantation and Translational Immunology, UMR 1064, ITUN, Nantes, France; Centre constitutif Filière OSCAR et Filière ORKID, CHU de Nantes, Nantes, France; Nephrogenetics Unit, Institute of Human Genetics, University Hospital Heidelberg, Heidelberg, Germany; Department of Medical Translational Sciences, University of Campania Luigi Vanvitelli, Naples, Italy; Biogem Scarl, Ariano Irpino, Italy; Department of Nephrology and Hypertension, IIS-Fundación Jiménez Díaz UAM, Madrid, Spain; Department of Medicine, RICORS2040, Madrid, Spain; Departamento de Medicina, Facultad de Medicina, Universidad Autónoma de Madrid, Madrid, Spain; Department of Nephrology, Research Institute for Medical Innovations and Radboud umc Center of Expertise for Rare Kidney Diseases, Radboud University Medical Center, Nijmegen, the Netherlands; Biosciences Institute, Newcastle University, Central Parkway, Newcastle upon Tyne, UK; Renal Services, The Newcastle upon Tyne NHS Foundation Trust, Newcastle upon Tyne, UK; National Institute for Health Research Newcastle Biomedical Research Centre, Newcastle upon Tyne, UK; Department de Néphrologie, Hémodialyse et Transplantation Rénale, Centre de référence MARHEA, Filière ORKID, CHRU Brest, Brest, France; Faculty of Pharmacy and Biochemistry, University of Zagreb, Croatia & Department of Nephrology and Dialysis, Dubrava University Hospital, Zagreb, Croatia; Department II of Internal Medicine, Faculty of Medicine and University Hospital, University of Cologne, Cologne, Germany; Center for Rare Diseases Cologne, Faculty of Medicine and University Hospital Cologne, University of Cologne, Cologne, Germany; Cologne Excellence Cluster on Cellular Stress Responses in Aging-Associated Diseases (CECAD), Cologne, Germany; Department of Nephrology and Medical Intensive Care, Charité - Universitätsmedizin Berlin, Berlin, Germany

**Keywords:** chronic kidney disease, family history, genetic kidney disease

## Abstract

Patients with chronic kidney disease often present with a family burden of disease. However, efficient collection and documentation of family history can be challenging in clinical practice. In this article, we provide 10 practical tips to support clinicians in obtaining family history information, with the goal of improving pre-stratification for genetic testing and guiding renal risk counselling for index patients and their relatives.

## TIPS

(1) Prepare the visit: inform the patient beforehand that family history will be an important topic.
(2) Take your time: family history admittedly takes time but is indispensable.
(3) Inquire individually rather than collectively: ask specific questions rather than inquiring whether everyone is fine in the family.
(4) Be specific, understandable, and rephrase your questions: make sure the patient understands what information is needed and why.
(5) Recognize variability in disease expressivity: it is hard to predict all information you are looking for, obtain as much information as possible without getting lost in the details.
(6) Tactfully explore consanguinity: consanguinity is a key but delicate and tricky question. Be aware of the cultural background.
(7) Be systematic and draw a pedigree: without a systematic approach key information is often lost and communication with colleagues difficult.
(8) Determine the inheritance pattern: from your pedigree drawings you will be able to better derive the expected pattern of inheritance.
(9) Facilitate screening in relatives and check validity: family appointments are often helpful for data completion.
(10) Obtain periodic updates: family history changes over time, and even more so the knowledge of patients on other affected family members.

Monogenic forms of kidney disease (MKD) were previously considered to be rare, however, recent studies have shown that at least 10%–30% of adults with chronic kidney disease (CKD) suffer from an inherited form of the disease [1–3]. Healthcare professionals are trained to inquire about familial diseases to assess cardiovascular risk factors or cancer, and most physicians accurately collect family histories in cases of well-recognized MKD such as autosomal dominant polycystic kidney disease or Alport syndrome. However, structured reporting of a family history of kidney disease is still lacking in many unexplained cases and evidence of inherited kidney disease may be missed due to an incomplete review of family history. Obtaining a thorough and meaningful family history is both an art and a time-intensive task, but it is crucial for guiding accurate diagnosis and determining the pre-test probability for genetic testing. While recessive diseases are more common in paediatric patients, clinicians are typically confronted with dominant inheritance patterns in cases of adult-onset disease. Therefore, family history becomes even more important in adult nephrology.

Assessment of family history has been found to be the most useful and cost-effective tool to estimate the risk for common chronic diseases [4]. Besides classical monogenic disease, family history also helps identifying situations of increased risk based on environmental factors or polygenic inheritance. With an estimated heritability of 44% and up to 30% of CKD patients reporting an affected family member [5], the systematic recording and documentation of family histories is essential, and ideally conducted in a standardized manner using an annotated pedigree diagram.

## 1. PREPARE THE VISIT

Before the consultation, if possible, consider sending a letter explaining that the patient's family history will be discussed as standard practice because inherited kidney diseases are prevalent. This advance notice allows patients to gather detailed medical histories from their relatives, including previous laboratory values with serum creatinine or urinary protein/albumin measurements, abdominal imaging, previous genetic testing, and any other significant medical information. This preparation can save valuable time during the consultation and also provides patients with the opportunity to psychologically prepare to share potentially painful details, such as the loss of children or other close family members. An example of such a letter is inserted in Appendix 1.

However, such preparation is not always feasible, and some healthcare professionals may prefer to initiate the discussion during the consultation. Regardless, it is crucial to begin the conversation about family history by explaining the importance of specific questions about family members. This helps patients understand the relevance of the questions, facilitates their cooperation, and ensures informative answers. Additionally, as part of the preparation, specific consent may be obtained from other family members to reveal sensitive information. Alternatively, in some countries, it is common practice to offer a pre-clinic consultation with a genetic counsellor to prompt initial patient consideration of family structure and details.

Example of an introduction sentence:

‘Kidney diseases can sometimes have a genetic origin it does not mean that it is more severe but it is important to assess this possibility to improve your care and eventually, care of your relatives. So today, we will explore your family's medical history. If you have the chance to speak to your family members beforehand, please make sure that they are fine with passing on potentially sensitive information. We will look not only at kidney disease but also at other conditions, as certain genetic disorders can manifest differently among family members. This comprehensive history might provide valuable insights into your own condition. It's okay if you don't know all the details—this is a common situation, and we’ll work with the information you have. Also, you may share information you would like to remain confidential.’

## 2. TAKE YOUR TIME

Collecting a thorough family history is a time-intensive but indispensable process. The tips at-hand aim to reduce the time efforts to a necessary minimum by suggesting a structured approach. Such an approach provides invaluable insights into the patient's personal history, life experiences, and challenges, all of which are crucial for the effective management of CKD. Establishing this record can also enhance the patient's comfort and cooperation. Investing time in this initial stage ultimately saves time in the long term by preventing potential complications and guiding more precise treatment. It is also important to acknowledge that not all information may be gathered in the first session, and this is a common and understandable occurrence (please see tip (10)). Consequently, documenting that information is still missing and should be obtained later on is also an important aspect.

## 3. INQUIRE INDIVIDUALLY RATHER THAN COLLECTIVELY

**Figure 1:**
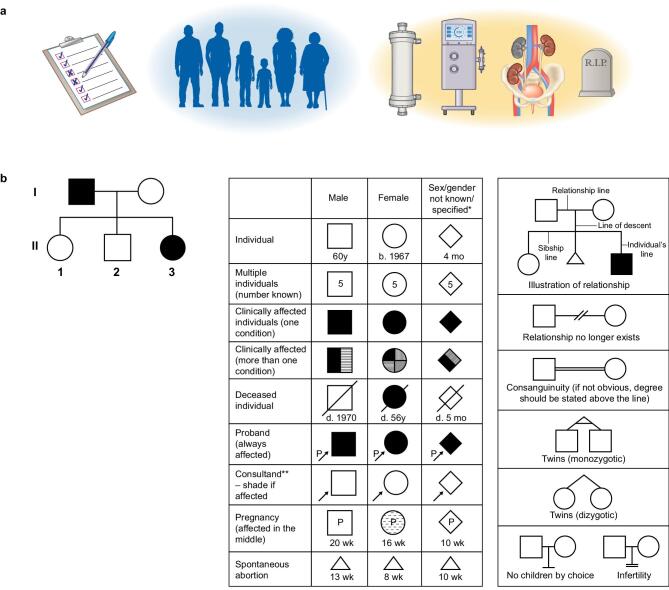
(**a**) Systematic collection of data on familial CKD burden requires standardized questions about renal replacement therapies (dialysis, kidney transplantation), as well as about sudden unexplained death and/or cause of death. (**b**) A minimum pedigree includes at least two generations. Notably, generations are labelled with Roman numerals (e.g. ‘I’ and ‘II’), starting with the older generation. Within each generation, individuals are numbered from left to right using Arabic numerals. Affected family members are depicted by filled symbols. More detailed pedigree annotations are shown in the box on the lower right.

Ask about each relative's medical history individually rather than collectively. Instead of asking, ‘Is everyone well?’ ask directly about specific conditions: ‘Have you heard of anyone in your family needing dialysis, kidney transplantation, or experiencing kidney failure?’ Ask specifically the same question pointing to siblings stating their names for instance, parents, children, etc. (Fig. 1a).

Also, inquire about other genetic diseases in the family, or any multisystem disease, or any specific follow up. Include both names and maiden names to help link individuals and identify previously ascertained cases.

## 4. BE SPECIFIC, UNDERSTANDABLE, AND REPHRASE YOUR QUESTIONS

Engage in targeted questioning to extract pertinent details; patients may not recognize what details are important. Adopt an investigative mindset akin to that of a detective, endeavouring to unveil crucial facts with each case. While patients are typically forthcoming, they may fail to grasp the significance of certain nuances. Use clear, lay language and ask targeted questions to gather precise data. For instance, in the case of an individual with CKD and a bland urinary sediment, where ADTKD is in the differential, it could be beneficial to explore the possibility of gout: a brief explanation of the term ‘gout’ coupled with specific inquiries about the presence of gout in family members can yield valuable insights.

It is imperative to rephrase questions effectively, as certain terms may not trigger relevant recollections or knowledge, while others might. For example, when inquiring about kidney disease, ask also if someone needed dialysis, or transplantation, as these life events are likely to be well-remembered. Also, do not forget to ask for the age of onset in the respective individual. Even if only roughly remembered, it is a valuable information. For extrarenal manifestation, list the relevant conditions depending on the context (e.g. hearing aids in Alport syndrome, gout in ADTKD). Patients may not recognize the importance of mentioning neurological or psychiatric conditions in their relatives, underscoring the necessity to clarify the specific information required.

Example of key questions:

‘What was the highest level of education completed by your mother/father?’
‘Do (did) you father/mother have high blood pressure?’
‘Do (did) you father/mother have gout? (and explain what gout is)’
‘Does anyone have hearing loss OR Does anyone wear hearing aids?’
‘Does any of your family member have autism? Schizophrenia? bipolar disorder?’

## 5. RECOGNIZE VARIABILITY IN DISEASE EXPRESSIVITY

Just like in the well-known parable ‘The blind men and the elephant’—originating from ancient Indian texts—each person describes only the part of the elephant they can touch—the full clinical picture only emerges when all aspects are considered. Understand that inherited kidney diseases are often syndromic and may present with a wide range of features that may vary within the same family. For instance, pathogenic variants in *HNF1B* may result in a single kidney in one member (unilateral kidney agenesis), diabetes mellitus in another, and an ADTKD-like presentation in a third. *PAX2*-related diseases can show as steroid-resistant nephrotic syndrome or focal segmental glomerulosclerosis, hypoplasia, a single kidney, or cystic dysplasia. Variations can cause clinicians to miss the diagnosis if they do not consider these diverse presentations. As a consequence, family history may be falsely labelled as ‘negative’ if variable expressivity and incomplete penetrance are not considered, or simply because of not making enough effort.

## 6. TACTFULLY EXPLORE CONSANGUINITY

Tactfully address the possibility of consanguineous unions. Depending on cultural or social backgrounds, this can be sensitive. Ask, ‘Is there a chance that your parents, grandparents, or great-grandparents might have had a common ancestor or were distant cousins?’ or ‘Are your parents related by blood?’ depending on the context. Inquire about the geographical origins of their parents to uncover potential consanguinity (e.g. ‘Do your parents come from the same village/same tribe?’).

## 7. BE SYSTEMATIC AND DRAW A PEDIGREE DIAGRAM

The most important tool for systematically documenting family histories is the pedigree. Pedigrees can be drawn together with patients and help ensure no family members are overlooked. Draw a pedigree chart that includes at least two generations (Fig. 1a) including the index case. Figure 1b summarizes the classical symbols and codes used to draw a pedigree. It is useful to comply with this guidance to facilitate reading and interpretation by other healthcare professionals (e.g. molecular biologists or clinical geneticists if the patient is subsequently referred for genetic counselling). Nonetheless, even an imperfect pedigree with non-standard symbols is preferable to the absence of any documentation, particularly if a legend is provided to clarify the symbols used. The fear of drawing an imperfect pedigree should not prevent anyone from collecting familial information.

It is generally helpful to have a routine when drawing a pedigree. For instance, starting with the siblings is sometimes easier than starting with children, as asking about children might trigger immediate genetic guilt or close the discussion if people have no children. Moving from generation to generation typically proves smoother than navigating disparate sections of the pedigree. This approach will also help you anticipate the space needed for the chart.

When asking about family members, it is important to specifically inquire about deceased relatives, as patients may not mention a family member who has passed away. Note the age and cause of death for each deceased individual, and include information about miscarriages and perinatal deaths. Record the age at onset of symptoms or the age when someone was considered unaffected, as well as the screening method used.

Finally, it is crucial to note ancestry and ask for parental consanguinity (see also tip (7)), given that certain kidney diseases exhibit higher prevalence within specific populations (such as *APOL1*-mediated kidney disease [6], ATTR-amyloidosis [7]) and recessive disorders are more common in consanguineous pedigrees [8].

Several online tools are available to create accurate pedigrees: some under licence and others are freely available (Table 1). The latter, however, often come without easy-to-use tutorials and may be non-intuitive for non-expert users. Another challenge concerns the electronic storage of pedigree data. Ideally, pedigrees are an integral part of the electronic health records, either by interface-based import from an external tool or via direct integration of drawing tools into the clinical information system.

**Table 1:** Examples of online tools and helpful resources to draw a pedigree.

Examples of online tools and helpful resources to draw a pedigree
Progeny: https://www.progenygenetics.com/online-pedigree/
Drawped: https://www.genecascade.org/ped-cgi/pedigree.cgi
https://humangenetics.medicine.uiowa.edu/resources/how-draw-pedigree
PhenoTips: https://phenotips.com
QuickPed: https://magnusdv.github.io/pedsuite/articles/web_only/quickped.html

Examples of key questions:

‘How many siblings do you have and did you have any siblings/children (depending on the context) who passed away?’

‘Do your siblings all have the same mother and father?’

## 8. DETERMINE INHERITANCE PATTERNS

Use the pedigree to evaluate the inheritance pattern if several family members are affected:

• Autosomal dominant: typically involves multiple generations with both males and females affected. However, please note, that negative family history does not exclude autosomal dominant disorders, e.g. due to de novo variants occurring in the index patient for the first time.
• Autosomal recessive: usually appears in siblings with both parents being carriers. More frequent in case of consanguineous unions.
• X-linked: typically, less severe presentation in affected females, with affected males not passing the condition to their sons.
• Mitochondrial inheritance: passed from mothers to all their children, regardless of sex. An affected male cannot pass the disease.

## 9. FACILITATE SCREENING IN RELATIVES AND CHECK YOUR SOURCES

To facilitate screening, it is helpful to offer family consultations with several family members coming to the outpatient clinic at a time. If there are paediatric patients among the screened ones, it is recommended to offer harmonized appointments together with paediatricians. For better ascertainment of siblings or children, prescribe simple examinations such as serum creatinine, UPCR (urinary protein/creatinine ratio), and abdominal ultrasound. Providing a clear screening plan can make it easier for asymptomatic individuals to get screened, instead of advising individual appointments. Document any prescriptions given on the pedigree. A so-called ‘family letter’ could be handed out to the index patient for providing information to all family members. This letter contains the information about the genetic diagnosis and the symptoms associated with it.

It is worth mentioning that certain information can be misleading. For instance, you may find that a relative described as ‘about to start dialysis’ by your patient actually has preserved kidney function but has been cautioned about the potential harm of diabetes if not managed properly. Similarly, a parent reported to have no cysts may not have undergone abdominal imaging to confirm this. Therefore, if relatives are willing to share their medical results, this results in an advantageous situation avoiding loss of or wrong interpretation of information.

## 10. OBTAIN PERIODIC UPDATES

Revisit and update the familial history periodically. Mark the date on your pedigree and review it regularly to note any new relatives (newborns, partners), diagnoses, deaths, or significant medical events. Regular updates ensure that the family history remains current and comprehensive. Use the electronic health record feature for automated recall of the medical history (when available): record taking the family history, a concise conclusion, the planned reassessment date, and where your pedigree file is stored, in the medical history section. This supports continuity of care during clinician handovers. Over time, phenotypes in relatives that were thought to be unaffected may be revealed and the pattern of inheritance may become more obvious.

Finally, if there is no family history of CKD, it does not rule out a genetic origin of the disease (*de novo* variant, variable expressivity, lack of phenotype/screening decades ago or depending on the geographical location, autosomal-recessive diseases, no info on family members etc.). However, any notion of family history is a strong and robust indicator for genetic testing, notably in CKD of unexplained cause (CKDx) [9].

## Data Availability

No new data were generated or analysed in support of this research.

## Appendix 1 APPENDIX 1: Example of letter that can be sent to the patient before the consultation

Dear Sir/Madam,

You are about to come for a consultation to discuss the cause of your kidney disease. In order to prepare you as well as possible for this consultation, we would like to inform you about the procedure and the important elements to prepare.

Preparation before the consultation: To get the most out of your consultation, it's a good idea to have the following items ready:

- A medical file listing your main medical history (feel free to bring any old letters or medical reports).
- A list of your current treatments.
- Your family history: Since genetic causes of kidney disease are common, obtaining more information on the medical history of your family is important. This includes information on the presence of diseases (not just kidney disease) in your parents, grandparents, uncles, aunts, cousins, and children. If members of your family have kidney disease, any information on the type and stage of kidney disease may be useful (from the presence of red blood cells/proteins in urine to a treatment by dialysis/kidney transplantation), and if they are being followed in another centre. It may be useful to ask them for the name of their nephrologist and their authorization to share medical information, from doctor to doctor according to national legislation. If your parents or grandparents or other relatives have died, please note their age at the time of death and the cause of death, if available.

You may want to keep some information private (such as miscarriage or adoption). These information may be beneficial to be known by your doctor, but can remains unwritten in your generalized medical chart if you want to. Do not hesitate to speak freely to your doctor and to express your wishes.

Questions and contacts: If you have any questions before your consultation, please do not hesitate to contact us at <<insert phone number>> <<insert e-mail address>>.

Thank you for your trust and cooperation. Your active participation and the preparation of this information are greatly appreciated and essential to our work.

## References

[1] KDIGO conference participants. Genetics in chronic kidney disease: conclusions from a Kidney Disease: Improving Global Outcomes (KDIGO) Controversies Conference. Kidney Int 2022;101:1126–41. 35460632 10.1016/j.kint.2022.03.019 PMC9922534

[2] Ottlewski I, Münch J, Wagner T et al. Value of renal gene panel diagnostics in adults waiting for kidney transplantation due to undetermined end-stage renal disease. Kidney Int 2019;96:222-230. 10.1016/j.kint.2019.01.038 31027891

[3] Wong K, Pitcher D, Braddon F et al. Effects of rare kidney diseases on kidney failure: a longitudinal analysis of the UK National Registry of Rare Kidney Diseases (RaDaR) cohort. Lancet 2024;403:1279–89. 10.1016/S0140-6736(23)02843-X 38492578 PMC11750427

[4] Ginsburg GS, Wu RR, Orlando LA. Family health history: underused for actionable risk assessment. Lancet 2019;394:596–603. 10.1016/S0140-6736(19)31275-9 31395442 PMC6822265

[5] Zhang J, Thio CHL, Gansevoort RT et al. Familial aggregation of CKD and heritability of kidney biomarkers in the general population: the Lifelines Cohort Study. Am J Kidney Dis 2021;77:869–78. 10.1053/j.ajkd.2020.11.012 33359149

[6] Genovese G, Friedman DJ, Ross MD et al. Association of trypanolytic *ApoL1* variants with kidney disease in African Americans. Science 2010;329:841–5. 10.1126/science.1193032 20647424 PMC2980843

[7] Delgado D, Dabbous F, Shivappa N et al. Epidemiology of transthyretin (ATTR) amyloidosis: a systematic literature review. Orphanet J Rare Dis 2025;20:29. 10.1186/s13023-025-03547-0 39819351 PMC11740649

[8] Hildebrandt F, Heeringa SF, Rüschendorf F. et al. A systematic approach to mapping recessive disease genes in individuals from outbred populations. PLos Genet 2009;5:e1000353. 10.1371/journal.pgen.1000353 19165332 PMC2621355

[9] Halbritter J, Figueres L, Van Eerde AM et al. Chronic kidney disease of unexplained cause (CKDx): a consensus statement by the Genes & Kidney Working Group of the ERA. Nephrol Dial Transplant 2025;gfaf092. 10.1093/ndt/gfaf092 40459916 PMC12709129

